# Prognostic value of preoperative circulating tumor DNA in non-small cell lung cancer: a systematic review and meta-analysis

**DOI:** 10.1007/s00432-023-05550-z

**Published:** 2024-01-22

**Authors:** Jiamin Lu, Yuqian Feng, Kaibo Guo, Leitao Sun, Shanming Ruan, Kai Zhang

**Affiliations:** 1 The First School of Clinical Medicine, Zhejiang Chinese Medical University, Hangzhou, Zhejiang China; 2 The First Affiliated Hospital of Zhejiang, Chinese Medical University, (Zhejiang Provincial Hospital of Chinese Medicine), Hangzhou, Zhejiang China; 3 Hangzhou TCM Hospital of Zhejiang Chinese Medical University, Hangzhou, Zhejiang China; 4 Department of Oncology, Affiliated Hangzhou First People’s Hospital, Zhejiang University School of Medicine, Hangzhou, Zhejiang China; 5 Anji Traditional Chinese Medical Hospital, Huzhou, Zhejiang China

**Keywords:** Preoperative circulating tumor DNA, Non-small-cell lung cancer, Adjuvant therapy, Meta-analysis, Systematic review

## Abstract

**Background:**

Several recent studies have reported the increasing application of preoperative circulating tumor DNA (ctDNA) as a biomarker of tumor burden for guiding potential postoperative treatment strategies.

**Methods:**

A meta-analysis of prospective/retrospective cohort studies was conducted to compare the prognosis of preoperatively genetically positive and genetically negative NSCLC patients. The endpoints used in the included studies were overall survival (OS) and recurrence-free survival (RFS). The objective of the meta-analysis was to comprehensively explore the prognostic value of preoperative ctDNA for patients with non-small-cell lung cancer (NSCLC) and its significance in guiding postoperative adjuvant therapy (AT) in patients with NSCLC.

**Results:**

The preliminary analysis identified 1565 studies, among which only 11 studies fulfilled the eligibility criteria and were finally included in the present systematic review and meta-analysis. The statistical results revealed that the expression of preoperative ctDNA was associated with worse RFS (HR = 3.00; 95% CI 2.26–3.98; *I*^2^ = 0%) and OS (HR = 2.77; 95% CI 1.67–4.58; *I*^2^ = 0%), particularly in lung adenocarcinoma (LUAD) patients (RFS: HR = 3.46; 95% CI 2.37–5.05; *I*^2^ = 0%; OS: HR = 3.52; 95% CI 1.91–6.49; *I*^2^ = 0%) and patients with I–II stage of NSCLC (RFS: HR = 2.84; 95% CI 1.88–4.29; *I*^2^ = 0%; OS: HR = 2.60; 95% CI 1.43–4.74; *I*^2^ = 0%). Moreover, compared to patients with negative preoperative ctDNA, patients with positive preoperative ctDNA presented greater survival benefits (HR = 0.39; 95% CI 0.22–0.67; *I*^2^ = 2%) from postoperative AT.

**Conclusion:**

The evaluation of the prognostic value of preoperative ctDNA revealed that preoperative ctDNA might be used as a prognostic biomarker for patients with LUAD or those with stage I–II NSCLC. In addition, postoperative AT is recommended for NSCLC patients with positive preoperative ctDNA, regardless of the disease stage and subtype.

**Supplementary Information:**

The online version contains supplementary material available at 10.1007/s00432-023-05550-z.

## Introduction

Lung cancer continues to be the primary cause of cancer-related mortality worldwide (Sung et al. 2021), and non-small-cell lung cancer (NSCLC) accounts for 85% of all cases of lung cancer (Siegel et al. 2017). Even after surgical resection and subsequent adjuvant therapy (AT), the risk of disease recurrence persists for several years among patients with stage I–III NSCLC (Isaka et al. 2018; Sawabata et al. 2011). Therefore, the identification of novel prognostic factors for patients with NSCLC is of great significance.

Recently, circulating tumor DNA (ctDNA) has been evaluated as a prognostic indicator of postoperative relapse and mortality in cancer (Benhaim et al. 2021; Hata et al. 2021) with great enthusiasm. The release of somatic DNA from tumor cells into the circulatory system upon shedding or apoptosis leads to the formation of ctDNA, and the significance of this tumor-specific biomarker has been demonstrated in numerous studies (Diaz and Bardelli 2014; Bettegowda et al. 2014; Diehl et al. 2005). Evidence suggests that postoperative positive ctDNA is correlated with the resurgence of NSCLC (Chen et al. 2019; Yang et al. 2020). In addition, preoperative ctDNA is reported to be considerably useful in predicting recurrence (Provencio et al. 2022; Gale et al. 2022). Preoperative ctDNA could better reflect the characteristics of the primary tumor, with a higher detection rate compared to postoperative ctDNA (Xia et al. 2022; Zhang et al. 2019). Therefore, discerning the preoperative ctDNA status could assist clinicians in identifying the higher risk of relapse and fatality in patients with NSCLC, thereby potentially altering the therapeutic approach used for these patients.

The multiple meta-analyses reported in recent years have assessed the prognostic implications of preoperative ctDNA detection in resectable NSCLC (Wang et al. 2022; Guo et al. 2022). The clinicopathological characteristics, such as ethnicity, pathological type, and stage, have also been reported as significant for treatment and prognosis (Ettinger et al. 2014). However, to the best of the author’s knowledge, the predictive value of preoperative ctDNA status in subgroups categorized based on the above characteristics remains to be determined so far.

Therefore, to highlight the significance of preoperative ctDNA in the precise diagnosis and treatment of patients with NSCLC, a comprehensive systematic review and meta-analysis was conducted to analyze the prognostic value of preoperative ctDNA in different subgroups (different races, pathological types, and stages) of these patients. In addition, the benefits of postoperative adjuvant therapy based on the preoperative ctDNA status were evaluated.

## Methods

### Study protocol

According to the Preferred Reporting Items for Systematic Reviews and Meta-Analyses (PRISMA) guidelines (Page et al. 2021), a systematic review of the literature and meta-analysis was conducted for patients with resected NSCLC to identify the respective relationships of preoperative ctDNA status with the survival outcomes, including relapse-free survival (RFS) and overall survival (OS). The present study is registered in the international prospective register of systematic reviews (PROSPERO 2022 CRD42022311615).

### Review of the literature

The electronic databases, including Cochrane Library, Embase, PubMed, and ScienceDirect, were searched for relevant literature published until 15 Jan 2023. The detailed search strategy is presented in Supplementary File 1. Both published articles and conference abstracts reported in all languages were included in the systematic review. Two authors (Kaibo Guo, Jiamin Lu) independently selected and examined the potentially relevant articles and abstracts based on the established eligibility criteria. Any disagreements were resolved through a discussion with another author (Kai Zhang).

### Study selection

The inclusion criteria for the potentially relevant studies were as follows: (1) observational studies (prospective or retrospective); (2) studies including patients with stage I–III NSCLC who underwent radical resection of any type; (3) studies that had recorded the preoperative ctDNA status using next-generation sequencing (NGS) and reported the corresponding outcome data in terms of either RFS, disease-free survival (DFS), progression-free survival (PFS), or OS. The exclusion criteria were as follows: (1) studies including patients with non-operative or stage IV NSCLC were excluded after a thorough reading of the full text or by analyzing raw data, (2) the authors, the clinical trial number, and the institutions mentioned in the text were examined to prevent the repeated inclusion of the same study.

### Data extraction and quality assessment

The following data were extracted from the included studies or related raw data: the year of publication, authors, the number of participants, study description, details of ctDNA detection, median follow-up duration, and survival outcomes, including RFS and OS. To ensure a thorough estimation of RFS, only the studies reporting outcome measures such as RFS, DFS, and PFS were included.

In the meta-analysis, preoperative ctDNA was considered a binary variable and classified into two groups (detected vs. not detected). Univariate and multivariate Cox regression analyses were conducted, based on which the survival effect size, including hazard ratio (HR) and 95% confidence interval (CI), was calculated from the raw data to the extent possible. If raw data were not provided in the study, the survival effect size was obtained by extracting the data reported in the studies or obtaining relevant information from the survival plots reported in the studies using the survival effect size software.

Newcastle–Ottawa Scale (NOS) was adopted to evaluate the quality of the included studies. In the NOS, a maximum of nine points were assessed, including the points of patient selection (4 points), outcome assessment (3 points), and comparability of the cohort (2 points) (Stang 2010).

### Data synthesis and main outcomes

A heterogeneity evaluation was conducted, and *I*^2^ was reported in all analyses. All HRs were pooled using both fixed- and random-effects models regardless of the degree of heterogeneity. In general, *I*^2^ > 50% or *P* value < 0.05 were considered to indicate heterogeneity.

In the fixed-effects model, the inverse variance method was adopted to calculate the overall HR. In the random-effects model, the DerSimonian–Laird method was adopted to determine heterogeneity. At *I*^2^ ≤ 50%, it was considered better to use a fixed-effects model to pool the HRs. On the other hand, a random-effects model was a better choice when *I*^2^ > 50%. The *P* values for the pooled HRs were not reported. Publication bias was detected through funnel plot analysis and Egger’s test. Leave-one-out sensitivity analyses were performed to assess the robustness of the findings. All analyses in the present study were performed using the R statistical software version 4.0.5 (R packages survival, Survminer, meta).

The primary endpoint of the present meta-analysis was the effect of preoperative ctDNA status on RFS and OS in patients with NSCLC.

The secondary endpoints were as follows: (1) preoperative ctDNA could predict the differences in RFS and OS between patients with lung adenocarcinoma (LUAD) and those with non-lung adenocarcinoma (non-LUAD); (2) tt is possible to predict the differences between patients with stage I–II and stage III NSCLC; and (3) the benefits of AT in NSCLC patients with positive or negative preoperative ctDNA status.

## Results

### Study selection

**Fig. 1:**
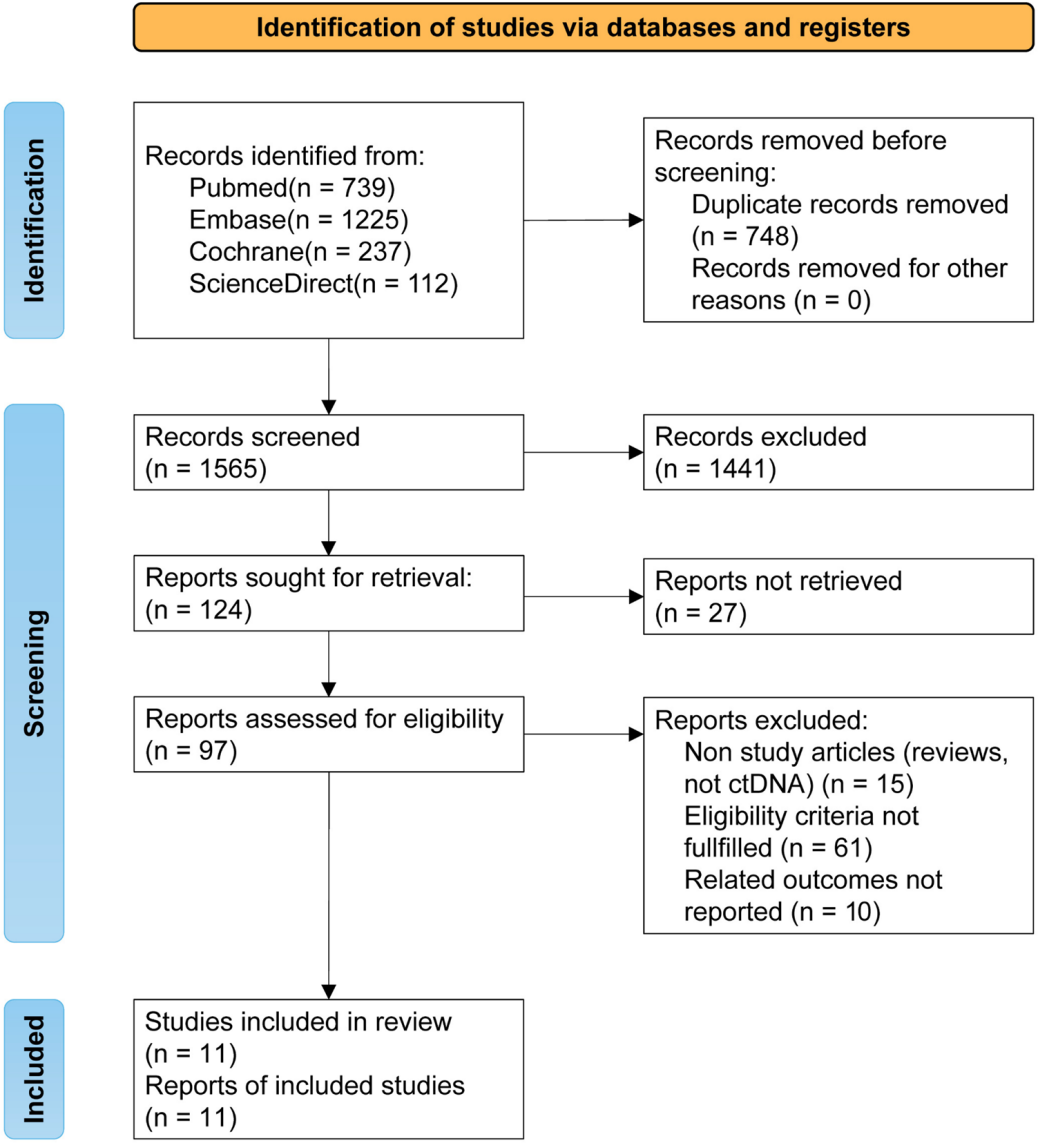
Literature search and study selection according to PRISMA 2020 flow diagram for systematic reviews

The literature search identified 1565 articles in total, among which 11 studies fulfilling the eligibility criteria were finally included in the present systematic review and meta-analysis. A flow chart of the screening process based on PRISMA is presented in Fig. 1.

### Characteristics of the included studies

Table 1 summarizes the participants and intervention characteristics of the studies included in the present meta-analysis. Table 2 details the ctDNA status and survival endpoint characteristics reported in the included studies.

**Table 1:** Study characteristics of the studies included in the systematic review and meta-analysis

Author	Country	Year	Non-small cell lung cancer subtype	Study description	Treatment in perioperative period	Stage distribution	NOS
Peng M	China	2020	LUAD *n* = 40 LUSC *n* = 30 Others *n* = 7	Prospective observational study (Ethics Committee of the Second Xiangya Hospital, Central South University, Project identification code: 2014S006)	The postoperative treatment: CT *n* = 14; RT *n* = 1; TT *n* = 3; CT + RT *n* = 3; CT + TT *n* = 3; CT + RT + TT *n* = 1; none *n* = 49; unknown *n* = 3	Stage I *n* = 40 Stage II *n* = 18 Stage III *n* = 17 Stage IV *n* = 2	8
Qiu B	China	2021	LUAD *n* = 60 LUSC *n* = 38 Others *n* = 5	Prospective observational study (ChiCTR1900024656)	The postoperative treatment: CT *n* = 72; TT *n* = 5; CT + RT *n* = 1; CT + TT *n* = 2; none *n* = 23	Stage I *n* = 12 Stage II *n* = 41 Stage III *n* = 48 Stage IV *n* = 2	7
Tan A	Singapore	2021	LUAD *n* = 48 Others *n* = 9	Prospective observational study	The postoperative treatment: CT *n* = 15; none *n* = 42	Stage I *n* = 39 Stage II n = 9 Stage III n = 9	5
Waldeck S	Germany	2022	LUAD *n* = 5 LUSC *n* = 10 Others *n* = 6	Prospective observational study (DRKS00009521)	The postoperative treatment: CT *n* = 9; RT *n* = 2; none *n* = 10	Stage I *n* = 2 Stage II *n* = 9 Stage III *n* = 10	7
Xia L	China	2022	LUAD *n* = 280 LUSC *n* = 43 Others *n* = 7	Prospective observational study (NCT03317080)	The postoperative treatment: CT *n* = 82; RT *n* = 1; TT *n* = 28; CT + RT *n* = 12; CT + TT *n* = 3; ICB *n* = 1; RT + TKI *n* = 1; CT + RT + TT *n* = 1; none *n* = 201	Stage I *n* = 221 Stage II *n* = 60 Stage III *n* = 49	9
Gale D	England	2022	LUAD *n* = 45 LUSC *n* = 21 Others *n* = 3	Prospective observational study (NCT04153526)	The postoperative treatment: CT = 2; RT = 1; CT + RT = 5; none *n* = 61	Stage I *n* = 42 Stage II *n* = 21 Stage III *n* = 6	8
Li N	China	2022	LUAD *n* = 87 LUSC *n* = 21 Others *n* = 11	Prospective observational study (NCT03465241)	The postoperative treatment: CT *n* = 34; none *n* = 85	Stage I *n* = 77 Stage II *n* = 24 Stage III *n* = 18	8
Yue D	China	2022	LUSC *n* = 14 Others *n* = 8	Prospective observational study (Ethics committee of Tianjin Medical University Cancer Institute and Hospital, Project identification code: E2020444A)	The preoperative treatment: nivolumab + platinum double chemotherapy *n* = 12; nivolumab + ipilimumab *n* = 4; docetaxel + cisplatin *n* = 4; pemetrexed + cisplatin *n* = 2	Stage I *n* = 5 Stage II *n* = 4 Stage III *n* = 13	6
Provencio M	Spain	2022	LUAD *n* = 24 LUSC *n* = 13 Others *n* = 4	Prospective observational study (NCT03081689)	The preoperative treatment: paclitaxel and carboplatin plus nivolumab *n* = 41; The postoperative treatment: nivolumab *n* = 37	Stage III *n* = 41	9
Chen K Z	China	2022	LUAD *n* = 73 LUSC *n* = 6 Others *n* = 2	Retrospectively observational study (China National Center for Bioinformation, Project identification code: HRA001278)	The preoperative treatment: None *n* = 81; The postoperative treatment: Unknow *n* = 81	Stage I *n* = 81	8
Zhang J T	China	2022	LUAD *n* = 203 LUSC *n* = 33 Others *n* = 25	Prospective observational study (Ethics committee of Guangdong Provincial People’s Hospital, Project identification code: 2018319H [R1])	The preoperative treatment: NAT *n* = 11; The postoperative treatment: AT *n* = 55	Stage I *n* = 163 Stage II *n* = 53 Stage III *n* = 45	7

*AT* adjuvant therapy, *ChiCTR* Chinese clinical trial registry, *CT* chemotherapy, *DRKS* deutsches register klinischer studien (German register of clinical trials), *ICB* immune checkpoint blockade, *ICI* immune checkpoint inhibitor, *LUAD* lung adenocarcinoma, *LUSC* lung squamous carcinoma, *NAT* neoadjuvant therapy, *NCT* national clinical trial, *NOS* Newcastle–Ottawa scale, *NSCLC* non-small cell lung cancer, *RT* radiation therapy, *TKI* tyrosine kinase inhibitor, *TT* targeted therapy

**Table 2:** Results of survival and ctDNA collections characteristics

Author	Country	Year	Number of pre-surgery patients (N)	Patients with evaluable ctDNA before operation (N)	Survival endpoints collected	Duration of follow-up	Method for ctDNA analysis	ctDNA positive criteria
Peng M	China	2020	77	75	OS, RFS	Median 44.0 months	127 gene cSMART (NGS & Multiplex-PCR)	Mutation ratio > 0
Qiu B	China	2021	103	87	RFS	Median 12.3 months	139 gene NGS panel and ATG-Seq	VAF ≥ 0.01%
Tan A	Singapore	2021	57	NA	RFS	Median 33.0 months	NGS and multiplex-PCR	NA
Waldeck S	Germany	2022	21	16	OS, RFS	Median 26.2 months	17 kb gene NGS panel (NGS and qPCR)	VAF ≥ 0.001%
Xia L	China	2022	330	330	RFS	Median 35.6 months	769 gene NGS panel	VAF ≥ 0.01%
Gale D	England	2022	69	65	OS, RFS	Median 18.1 months	NGS and multiplex-PCR	VAF ≥ 0.0001%
Li N	China	2022	119	117	OS, RFS	Median 30.7 months	425 gene NGS panel	VAF ≥ 1%
Yue D	China	2022	22	22	RFS	Median 17.7 months	194 gene NGS panel	VAF ≥ 0.3%
Provencio M	Spain	2022	41	40	OS, RFS	Median 38.0 months	409 gene NGS panel	MAF ≥ 0.1%
Chen K Z	China	2022	81	55	OS, DFS	Median 62.0 months	457 gene NGS panel	VAF > 0.1%
Zhang J T	China	2022	261	261	RFS	Median 19.7 months	338 gene NGS panel	VAF ≥ 0.01%

*ATG-Seq* automated triple groom sequencing, *cSMART* circulating single-molecule amplification and resequencing technology, *MAF* minor allele frequency, *Multiplex-PCR* multiplex polymerase chain reaction, *NA* not available, *NGS* next-generation sequencing, *OS* overall survival, *qPCR* quantitative real-time polymerase chain reaction, *RFS* recurrence-free survival, *VAF* variant allele frequency, *ctDNA* circulating tumor DNA, *LUAD* lung adenocarcinoma, *NSCLC* non-small cell lung cancer

Among the 11 prospective observational studies selected for the present meta-analysis, 4 studies (Provencio et al. 2022; Gale et al. 2022; Tan et al. 2021; Waldeck et al. 2022) were conducted with the European population and 7 studies (Xia et al. 2022; Peng et al. 2020; Qiu et al. 2021; Li et al. 2022; Zhang et al. 2022; Yue et al. 2022; Chen et al. 2022) were conducted with the Asian population. Irrespective of the pathological types of NSCLC, LUAD accounted for a significant proportion (73%) in the study population, while lung squamous carcinoma (LUSC) accounted for just 20%. In regard to NSCLC staging, Provencio et al. (2022) focused only on stage III NSCLC patients, Chen et al. (2022) limited their evaluation to patients with stage I NSCLC, and the remaining nine studies (Gale et al. 2022; Xia et al. 2022; Tan et al. 2021; Waldeck et al. 2022; Peng et al. 2020; Qiu et al. 2021; Li et al. 2022; Zhang et al. 2022; Yue et al. 2022) included patients with stage I–III NSCLC. In addition to surgery, neoadjuvant therapy (NAT) was stated in the full text of three studies (Provencio et al. 2022; Zhang et al. 2022; Yue et al. 2022), among which the study of Zhang et al. (2022) was excluded due to a lack of detailed data. Postoperative AT was stated in the full text of ten studies (Provencio et al. 2022; Gale et al. 2022; Xia et al. 2022; Tan et al. 2021; Waldeck et al. 2022; Peng et al. 2020; Qiu et al. 2021; Zhang et al. 2022; Li et al. 2022; Chen et al. 2022), among which four studies (Gale et al. 2022; Xia et al. 2022; Waldeck et al. 2022; Qiu et al. 2021) were finally included in the meta-analysis based on the availability of raw data. The survival effect size was calculated using the raw data from six studies (Xia et al. 2022; Gale et al. 2022; Waldeck et al. 2022; Peng et al. 2020; Qiu et al. 2021; Chen et al. 2022) and the survival plot information was extracted from Li et al. (2022) using the survival effect size software.

NGS was used for ctDNA analysis in all studies, and Provencio et al. (2022) defined minor allele frequency (maf) ≥ 0.1% as the criterion for ctDNA positivity. Eight studies (Gale et al. 2022; Xia et al. 2022; Waldeck et al. 2022; Qiu et al. 2021; Li et al. 2022; Zhang et al. 2022; Yue et al. 2022; Chen et al. 2022) selected variant allele frequency (vaf) greater than a certain threshold as the standard for measuring ctDNA positivity, among which Qiu et al. (2021) defined vaf ≥ 0.01% as the standard for preoperative ctDNA positivity, while Tan et al. (2021) and Peng et al. (2020) did not report this kind of standard.

The definitions of RFS and OS in the included trials are provided in Supplementary Table 1. The specific NOS scores for each study are presented in Supplementary Table 2. The combined results of univariate analyses are presented in Supplementary Table 3. The combined results of the multivariate analyses are presented in Supplementary Table 4.

### Association of preoperative ctDNA with RFS and OS

**Fig. 2:**
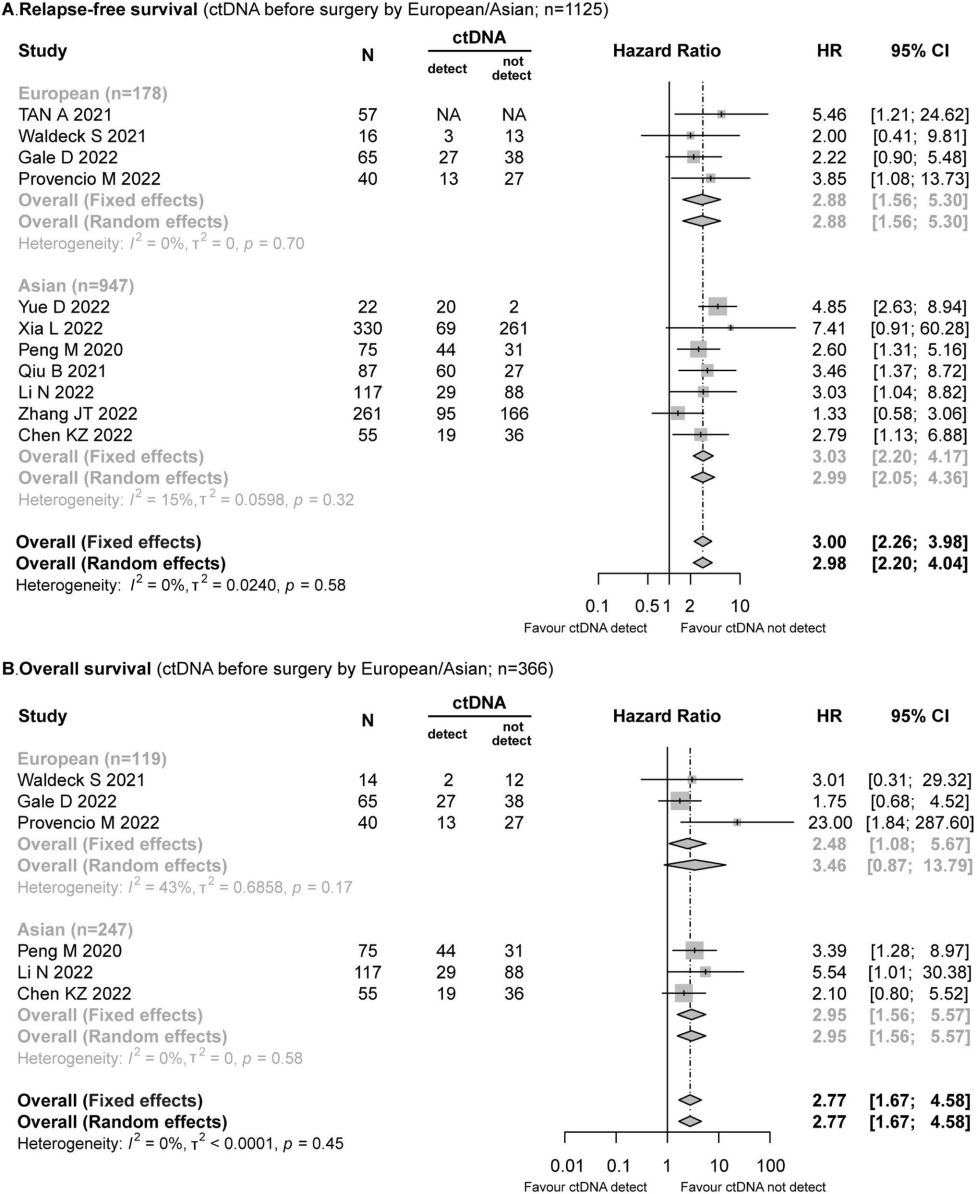
Forest plot for the impact of preoperative ctDNA in European/Asians with NSCLC. **A** Relapse-free survival. **B** Overall survival

A total of 11 studies (*n* = 1125) recorded the data of preoperative ctDNA status and RFS, which included the studies conducted with Europeans (*n* = 178) (Provencio et al. 2022; Gale et al. 2022; Tan et al. 2021; Waldeck et al. 2022) and Asians (*n* = 947) (Xia et al. 2022; Peng et al. 2020; Qiu et al. 2021; Li et al. 2022; Zhang et al. 2022; Yue et al. 2022; Chen et al. 2022). The patients with NSCLC who tested ctDNA positive prior to surgery exhibited a significantly higher risk of relapse (HR = 3.00; 95% CI 2.26–3.98; *I*^2^ = 0%). Moreover, these results (Fig. 2A) were similar in both European (HR = 2.88; 95% CI 1.56–5.30; *I*^2^ = 0%) and Asian (HR = 3.03; 95% CI 2.20–4.17; *I*^2^ = 15%) populations.

Six studies (*n* = 366) provided data on OS (Provencio et al. 2022; Gale et al. 2022; Peng et al. 2020; Li et al. 2022; Chen et al. 2022). Among these studies, ctDNA was detected in 42/119 (35.29%) of Europeans (Provencio et al. 2022; Gale et al. 2022; Waldeck et al. 2022) and 92/247 (37.25%) of Asians (Peng et al. 2020; Li et al. 2022; Chen et al. 2022), and positive ctDNA was associated with worse OS (HR = 2.77; 95% CI 1.67–4.58; *I*^2^ = 0%). In Europeans, the risk of death in patients with positive ctDNA was 1.48 times higher than that in the patients with negative ctDNA, and a similar trend was observed in the Asian populations (HR = 2.95; 95% CI 1.56–5.57; *I*^2^ = 0%) (Fig. 2B). In the leave-one-out meta-analysis, the overall results remained similar (Supplementary Fig. 1).

In two studies (Provencio et al. 2022; Yue et al. 2022), the ctDNA detection time was from the end of NAT to the preoperative time point. The ctDNA was detected in 33/62 (53.23%) patients, who were also revealed to be prone to experiencing a worse RFS (HR = 4.59; 95% CI 1.55–13.61; *I*^2^ = 0%) (Supplementary Fig. 2). OS analysis was not conducted due to a lack of sufficient data.

### Survival impact of preoperative ctDNA on LUAD and non-LUAD

**Fig. 3:**
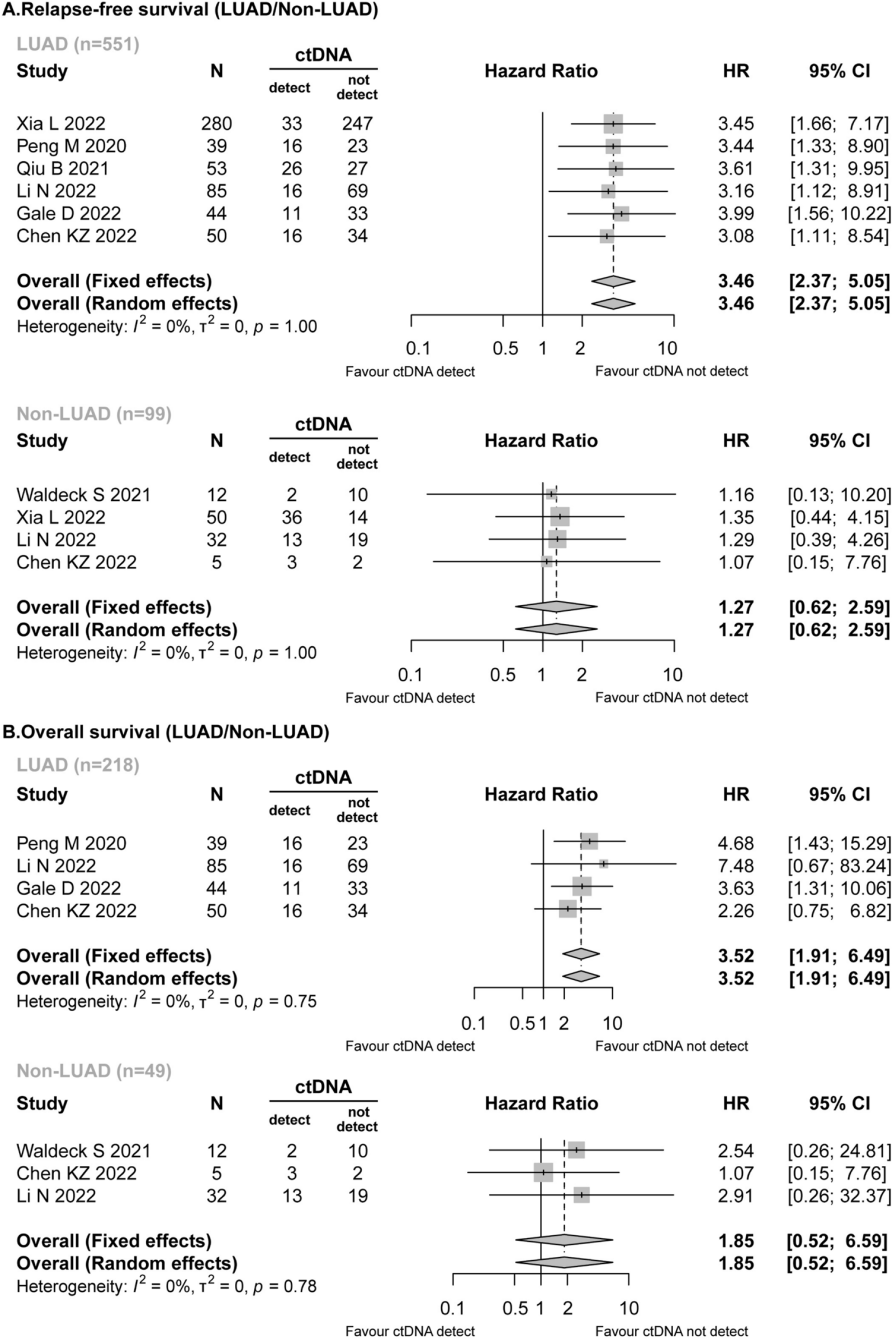
Forest plot for the impact of preoperative ctDNA in LUAD/non-LUAD patients. **A** Relapse-free survival. **B** Overall survival

Seven studies (Gale et al. 2022; Xia et al. 2022; Waldeck et al. 2022; Peng et al. 2020; Qiu et al. 2021; Li et al. 2022; Chen et al. 2022) reported detailed data on the pathological types of NSCLC. In the RFS analysis, preoperative ctDNA was detected in both LUAD patients (118/551, 21.42%) and non-LUAD patients (54/99, 54.55%). In the LUAD patients, the detected preoperative ctDNA was revealed to be associated with worse RFS (HR = 3.46; 95% CI 2.37–5.05; *I*^2^ = 0%), while for patients with non-LUAD, preoperative ctDNA positivity did not have a significant effect on RFS (HR = 1.27; 95% CI 0.62–2.59; *I*^2^ = 0%) (Fig. 3A).

Similarly, the presence of pre-surgery ctDNA was significantly related to shorter OS in LUAD patients (HR = 3.52; 95% CI 1.91–6.49; *I*^2^ = 0%), while a different result was obtained for the non-LUAD patients (HR = 1.85; 95% CI 0.52–6.59; *I*^2^ = 0%) (Fig. 3B).

### Survival outcomes of preoperative positive ctDNA in specific stages of the disease

**Fig. 4:**
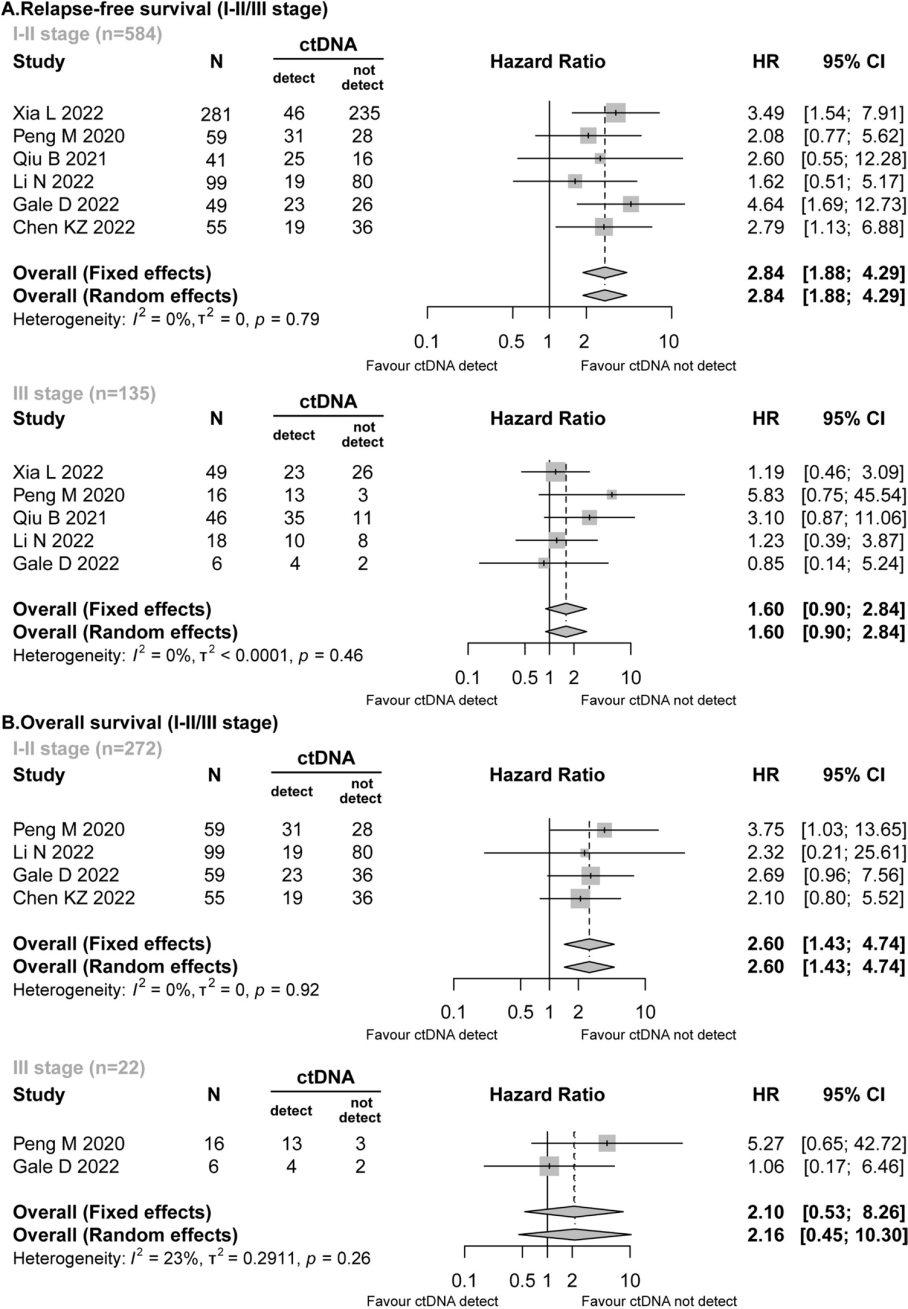
Forest plot for the impact of preoperative ctDNA in patients with stage I–II/stage III NSCLC. **A** Relapse-free survival. **B** Overall survival

Six studies (Gale et al. 2022; Xia et al. 2022; Peng et al. 2020; Qiu et al. 2021; Li et al. 2022; Chen et al. 2022) reported detailed data on the stage of NSCLC. In the RFS analysis, preoperative ctDNA was detected in patients with stage I–II (163/584, 27.91%) and those with stage III (85/135, 62.96%). Stage I–II patients with positive preoperative ctDNA presented worse RFS (HR = 2.84; 95% CI 1.88–4.29; *I*^2^ = 0%), while stage III patients did not exhibit a statistically significant difference in preoperative ctDNA positivity (HR = 1.60; 95% CI 0.90–2.84; *I*^2^ = 0%) (Fig. 4A).

Similarly, in the OS analysis, the presence of preoperative ctDNA was associated with a significantly higher risk of mortality in patients with stage I–II (HR = 2.60; 95% CI 1.43–4.74; *I*^2^ = 0%). However, no comparable association was observed in patients with stage III NSCLC (HR = 2.10; 95% CI 0.53–8.26; *I*^2^ = 23%) (Fig. 4B).

### Effects of adjuvant therapy on patients with positive or negative preoperative ctDNA status

**Fig. 5:**
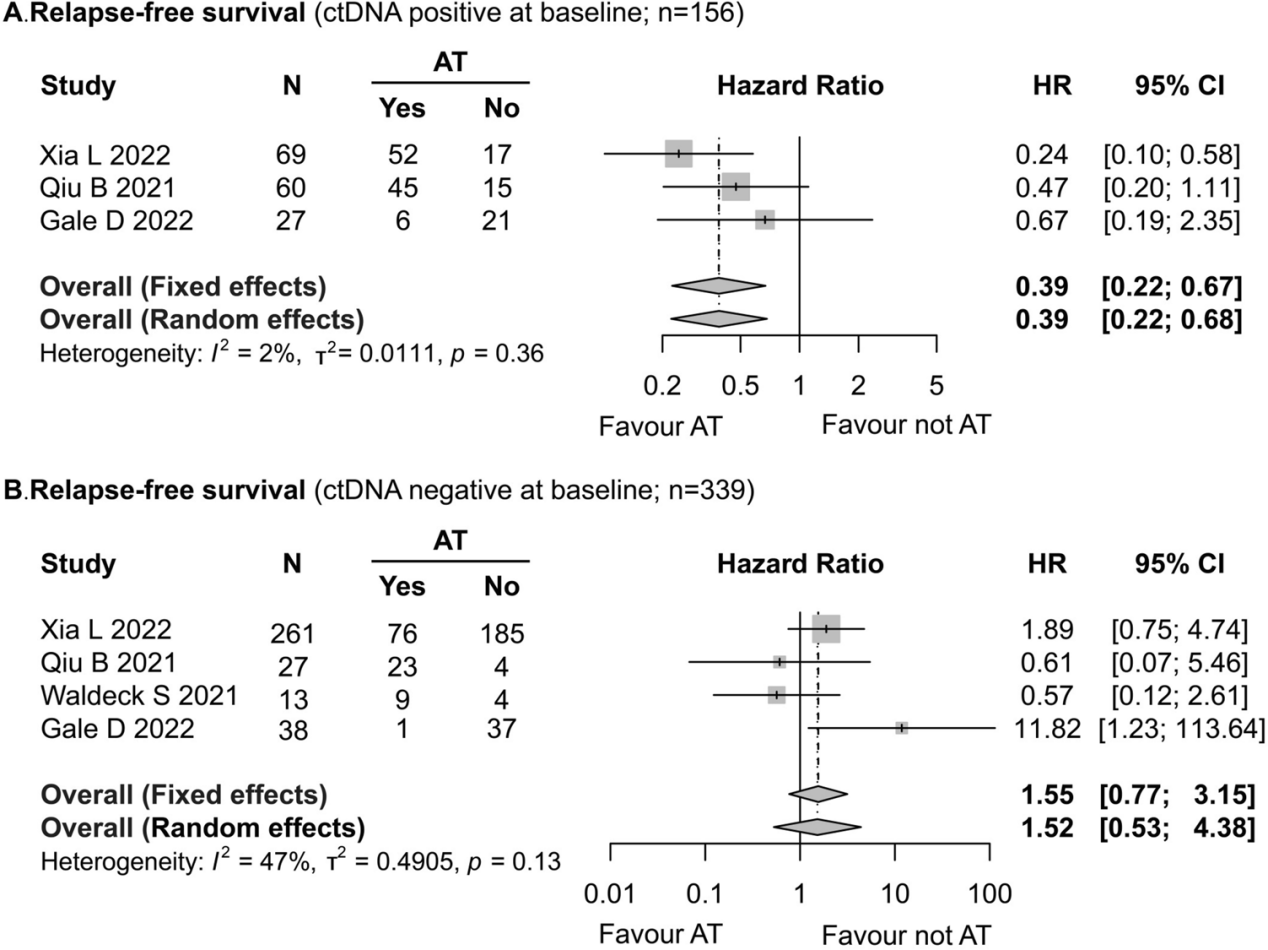
Forest plot of the impact of postoperative adjuvant therapy on recurrence-free survival in NSCLC patients. **A** Positive preoperative ctDNA. **B** Negative preoperative ctDNA

Four studies (Gale et al. 2022; Xia et al. 2022; Waldeck et al. 2022; Qiu et al. 2021) included in the present meta-analysis reported the effects of postoperative AT on RFS in two groups of patients with NSCLC (patients with preoperative ctDNA positive or negative). The patients with positive preoperative ctDNA (103/156, 66.03%) underwent AT postoperatively, and these patients presented better RFS (HR = 0.39; 95% CI 0.22–0.67; *I*^2^ = 2%) (Fig. 5A), while AT did not significantly improve RFS in the NSCLC patients with negative preoperative ctDNA (HR = 1.55; 95% CI 0.77–3.15; *I*^2^ = 43%) (Fig. 5B). The difference between preoperative ctDNA positive and negative patients in terms of the effects of AT on OS could not be compared due to lack of data.

### Quality estimation and the risk of bias analysis

**Fig. 6:**
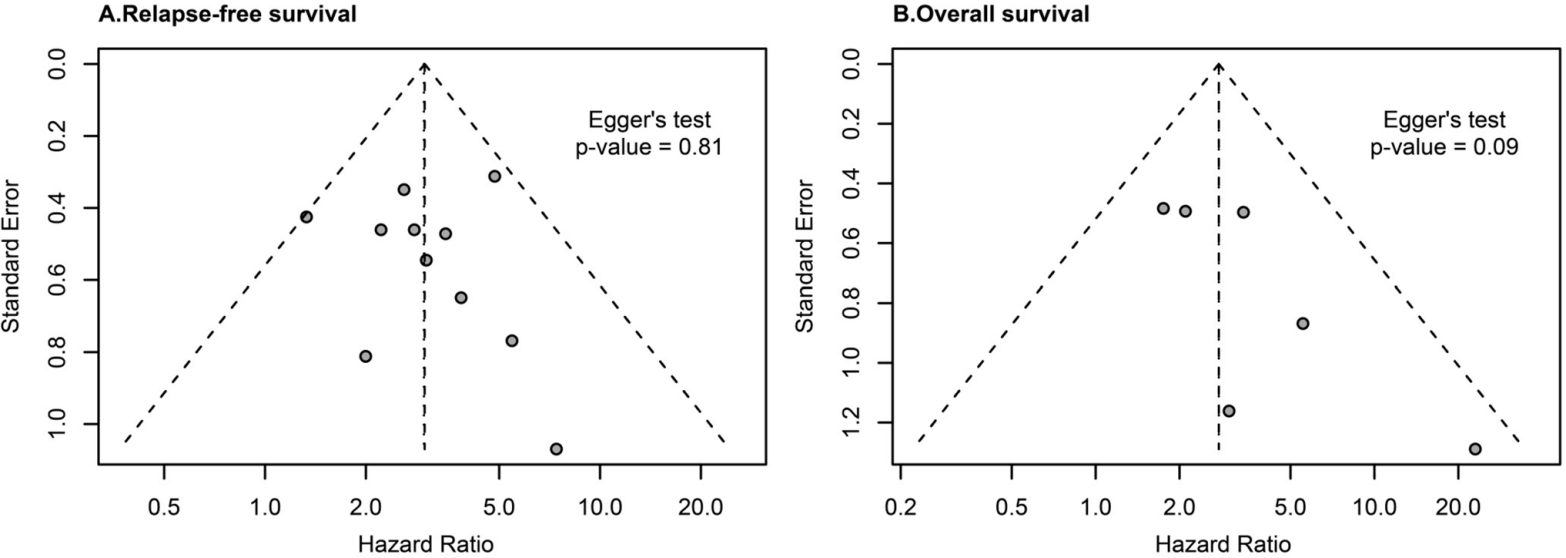
Fixed-effects funnel plots for the risk of publication bias. **A** Relapse-free survival. **B** Overall survival. The *P* value was calculated by the Egger’s test

All studies used NOS scoring in the range of 5–9 points. No publication bias was revealed in the studies included in the RFS analysis (Egger’s test = 0.81) and those included in the OS analysis (Egger’s test = 0.09) (Fig. 6). The summary of publication bias of the remaining studies is presented graphically in Supplementary Fig. 3A–J, and similar to the above studies, no indication of any publication bias was observed.

## Discussion

Recently, an increasing number of studies have been reporting the effectiveness of using elevated postoperative ctDNA levels as a significant indicator of cancer recurrence and death (Chen et al. 2019; Yang et al. 2020), which is attributed to the close association of ctDNA with postoperative residual tumor (Nakamura et al. 2021). However, if the patients who have undergone surgical resection continue to have a relatively small amount of cancer cells, postoperative ctDNA status cannot be positive, which leads to a decrease in the detection rate of ctDNA after surgery. Qiu et al. (2021) recorded that the ctDNA positivity rate of NSCLC patients decreased from 69.3% preoperatively to 21.2% postoperatively, which suggested the sensitivity of preoperative ctDNA could be higher than the sensitivity of postoperative ctDNA. Although ctDNA positivity certainly suggested a risk of cancer recurrence and death, Fakih et al. (2022) reported that a negative status of postoperative ctDNA would be common in low-volume metastatic disease, particularly in the metastatic disease of the lung. Therefore, determining the preoperative ctDNA status could assist in identifying a greater number of patients with a high risk of recurrence and death. Furthermore, preoperative ctDNA could better reflect the situation of the primary tumor compared to postoperative ctDNA in patients with resectable NSCLC based on the postoperative heterogeneity of the tumor (Saber et al. 2017).

Polymerase chain reaction (PCR) and next-generation sequencing (NGS) are usually employed to detect and quantify ctDNA. NGS is a high-throughput technical platform that may be employed to detect multiple genes simultaneously, with high sensitivity and accuracy (Sussman et al. 2020). Eunhyang Park et al. (Park and Shim 2020) compared the results obtained using several detection methods, such as NGS, PCR, and fluorescence in situ hybridization, in patients with lung cancer and reported that NGS could detect false negatives in PCR along with certain additional genetic mutations, which could be useful in guiding the implementation of interventions based on targeted drugs. Vanderpoel et al. (2022) assessed the total cost of NGS testing versus PCR detection among NSCLC patients and reported that compared to PCR testing of newly diagnosed NSCLC patients, NGS exhibited a rapid initiation of the appropriate targeted therapy and a lower cost of detection overall. These results indicated that NGS might become the mainstream method of ctDNA testing and analysis in the future.

Several recent meta-analyses have evaluated the clinical relevance of ctDNA in NSCLC patients. Guo et al. (2022) assessed the pooled sensitivity and specificity of ctDNA in the detection of minimal residual disease (MRD) and discovered that positive ctDNA was associated with an unfavorable prognosis in patients with localized NSCLC. Wang et al. (2022) assessed the prognostic potential of ctDNA detection at different time points in patients with resectable NSCLC and demonstrated that ctDNA detection between 3 days and 2 weeks after surgery had greater reliability and feasibility in identifying patients with resectable NSCLC who were at a higher risk for recurrence. The present study involved a further comprehensive analysis of the effects of preoperative ctDNA mutations detected using NGS techniques on the survival outcomes of NSCLC patients with different clinicopathological characteristics. Among the studies included in the present meta-analysis, ctDNA detection was used and reported more frequently in Asian populations than in European populations (Zhang et al. 2021). However, the European population had a higher incidence of lung cancer compared to the non-Europeans, according to Cancer Research UK (Delon et al. 2022). When a subgroup analysis of these populations was conducted in the present meta-analysis, it was revealed that preoperative ctDNA had a credible prognostic value in both Europeans and Asians. In addition, the following results were revealed: (1) elevated preoperative ctDNA level could serve as a prognostic factor for recurrence and death in LUAD patients, although it did not significantly predict survival (different kinds) in non-LUAD patients; (2) elevated preoperative ctDNA levels were associated with shorter RFS and OS in patients with stage I–II NSCLC while having no significant prognostic significance for patients with stage III NSCLC; (3) postoperative AT significantly improved RFS in NSCLC patients with positive preoperative ctDNA and not in those with negative preoperative ctDNA.

The Cox regression analysis revealed that the pathological types and clinical stages were important factors affecting the survival outcomes of patients with NSCLC. First, the preoperative ctDNA was analyzed in NSCLC patients with different pathological types. It was revealed that preoperative ctDNA detection was related to the risk of recurrence and death in LUAD patients and not in non-LUAD patients. This could be due to the heterogeneity of LUAD and LUSC, as in the subgroup of non-LUAD patients, patients with LUSC accounted for 84.5% of the total number of patients (71/84). Hematogenous metastasis is a prominent characteristic of early-stage LUAD (Gu et al. 2022; Kaseda et al. 2013), and although the distant metastasis rate of LUSC is lower than that of LUAD (Kelsey et al. 2009), its local recurrence, including that in the lymph nodes, is more frequent (Ikemura et al. 2017). In the studies included in the present meta-analysis, plasma-derived ctDNA was detected and reported to have a greater association with hematogenous metastasis in NSCLC compared to local metastasis. As a consequence, the rate of false-negative outcomes associated with preoperative ctDNA in predicting relapse and mortality in patients diagnosed with LUSC is higher compared to that of LUAD patients. Therefore, when using preoperative ctDNA detection alone, LUSC recurrence and death were likely to be missed, which could be the reason for preoperative ctDNA not resulting in a significant prognosis in non-LUAD patients. In summary, preoperative detectable levels of ctDNA were associated with disease burden and risk of recurrence in patients with LUAD, while this evaluation in LUSC patients lacked accuracy. Therefore, preoperative ctDNA testing is recommended for patients with LUAD.

In the present meta-analysis, preoperative ctDNA detection was revealed to be related to the risk of recurrence and death in patients with stage I–II NSCLC, while no significant association was observed in stage III patients, which is probably because stage III cases represent a heterogeneous group (Allen and Jahanzeb 2008), with a 5-year OS in the range of 15–35% for stage IIIA disease and 5–10% for stage IIIB (Burdett et al. 2007). Interestingly, it was revealed that the specificity of preoperative ctDNA in predicting recurrence and death in patients with stage III NSCLC was lower than that in patients with stage I–II NSCLC (Xia et al. 2022; Qiu et al. 2021). Therefore, positive preoperative ctDNA might not provide a reliable prediction of an unfavorable prognosis for patients with stage III NSCLC. Therefore, preoperative ctDNA detection is recommended for monitoring recurrence in patients with resected NSCLC of stage I–II.

Currently, increasing evidence suggests that postoperative AT could be used for preventing recurrence in operative NSCLC patients. However, patients with the IA stage have not benefitted from AT (Morgensztern et al. 2016), and the effect of AT on the IB stage patients remains debatable so far (Artal Cortés et al. 2015). A previously reported meta-analysis of 26 studies discovered that postoperative ACT could improve the 5-years OS in approximately 4% of the patients with NSCLC (Burdett et al. 2015), indicating that the effect of AT was much less than that of surgical resection. Therefore, further research is warranted to identify novel prognostic factors that would enable predicting which NSCLC patients would benefit from postoperative AT. In the present meta-analysis, patients with positive preoperative ctDNA who underwent AT postoperatively, were associated with better RFS, while it was revealed that AT did not significantly improve RFS in the NSCLC patients with negative preoperative ctDNA. Thus, it can be deduced that ctDNA detection can aid in the identification of NSCLC patients who are at a heightened risk of recurrence. This identification can enable the administration of appropriate treatment, such as AT, to maximize therapeutic benefits, potentially avoiding the need for unnecessary treatments in patients with negative preoperative ctDNA.

As with all research, the present meta-analysis also has certain limitations. First, preoperative ctDNA was used as a binary variable (detected/undetected) and could be easily extracted from the studies. However, it is noteworthy that the literature reports the use of considerable diversity of ctDNA analysis methodologies, and the distinct driving mutations could dictate the prognostic outcome, which was not evaluated in the present work. Second, a composite endpoint referred to as RFS was used in the present work in place of the RFS, PFS, and DFS endpoints reported in the included studies, although it must be acknowledged that all of these might not be identical in all aspects. Third, only 11 studies were included in the present work, a few of which had a relatively small sample size. As a result, survival effect size could not be calculated for certain studies by extracting data from these studies. While the results were significant, the number of trials in the neoadjuvant and stage subgroups of NSCLC was insufficient. Fourth, it must be admitted that the funnel plot may not detect publication bias when the number of studies is small. Therefore, the possibility that the available evidence could be limited and insufficient for definitive conclusions must be considered.

In summary, the present meta-analysis revealed a correlation between the presence of preoperative ctDNA and long-term prognosis in patients with NSCLC, particularly those diagnosed with LUAD or with a disease of clinical stages I–II. Moreover, the findings suggested that NSCLC patients with positive preoperative ctDNA could derive substantial survival benefits from AT. Appropriate incorporation of preoperative ctDNA detection in the treatment strategy of NSCLC patients could assist in identifying the cases with a risk of relapse, thereby being useful in guiding the postoperative treatment strategies formulated for these patients. However, the present meta-analysis also raises several questions regarding the application of preoperative ctDNA in NSCLC patients, which have to be addressed in future multicenter, large-sample-size, high-quality clinical trials.

## Supplementary Information

Below is the link to the electronic supplementary material.

Supplementary file1 (PDF 197 KB)

Supplementary file2 (PDF 169 KB)

Supplementary file3 (PDF 326 KB)

Supplementary file4 (PDF 103 KB)

Supplementary file5 (PDF 713 KB)

## Data Availability

The datasets generated during and/or analyzed during the current study are available from the corresponding author on reasonable request.
