# Supplementary figures and images for: Prognostic value of preoperative circulating tumor DNA in non-small cell lung cancer: a systematic review and meta-analysis
Source: J Cancer Res Clin Oncol. 2024 Jan 22;150(1):25. doi: 10.1007/s00432-023-05550-z (PMC10803397; doi:10.1007/s00432-023-05550-z)

Supplementary Figure 1. Leave-one-out analysis

A.Relapse-free survival

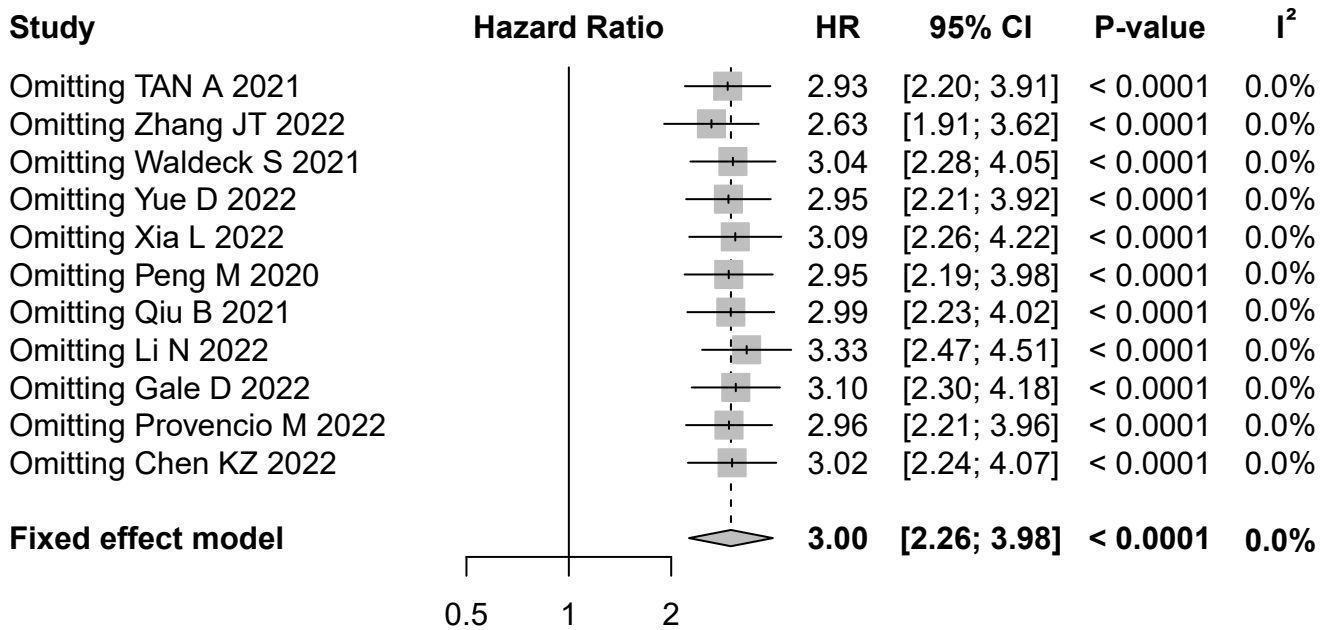

B.Overall Survival

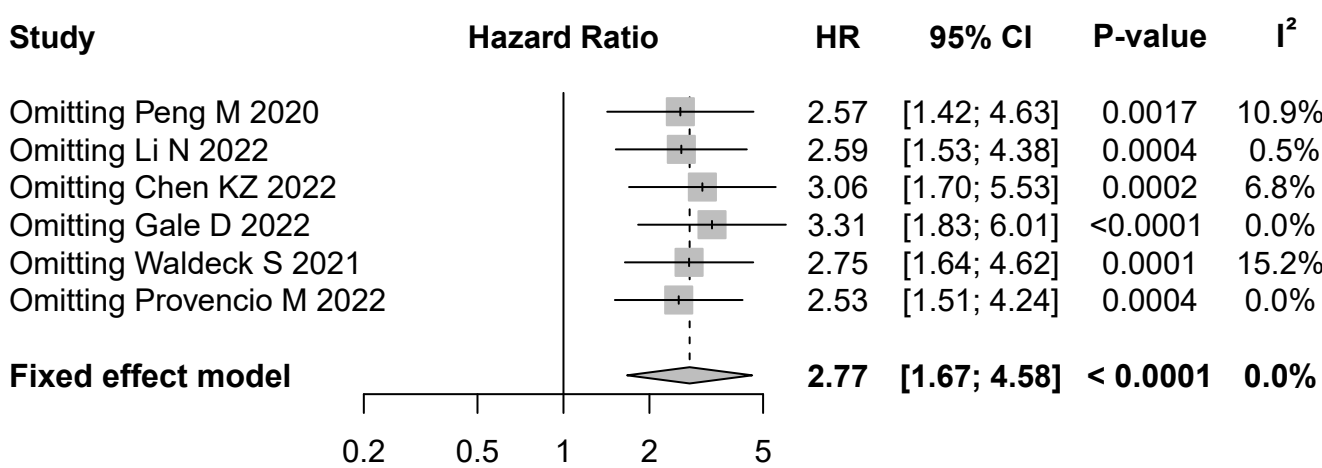

Supplement: Supplementary file 1 — Supplementary file1 (PDF 197 KB) [file 432_2023_5550_MOESM1_ESM.pdf]

Supplementary Figure 3. Funnel plots and Egger tests

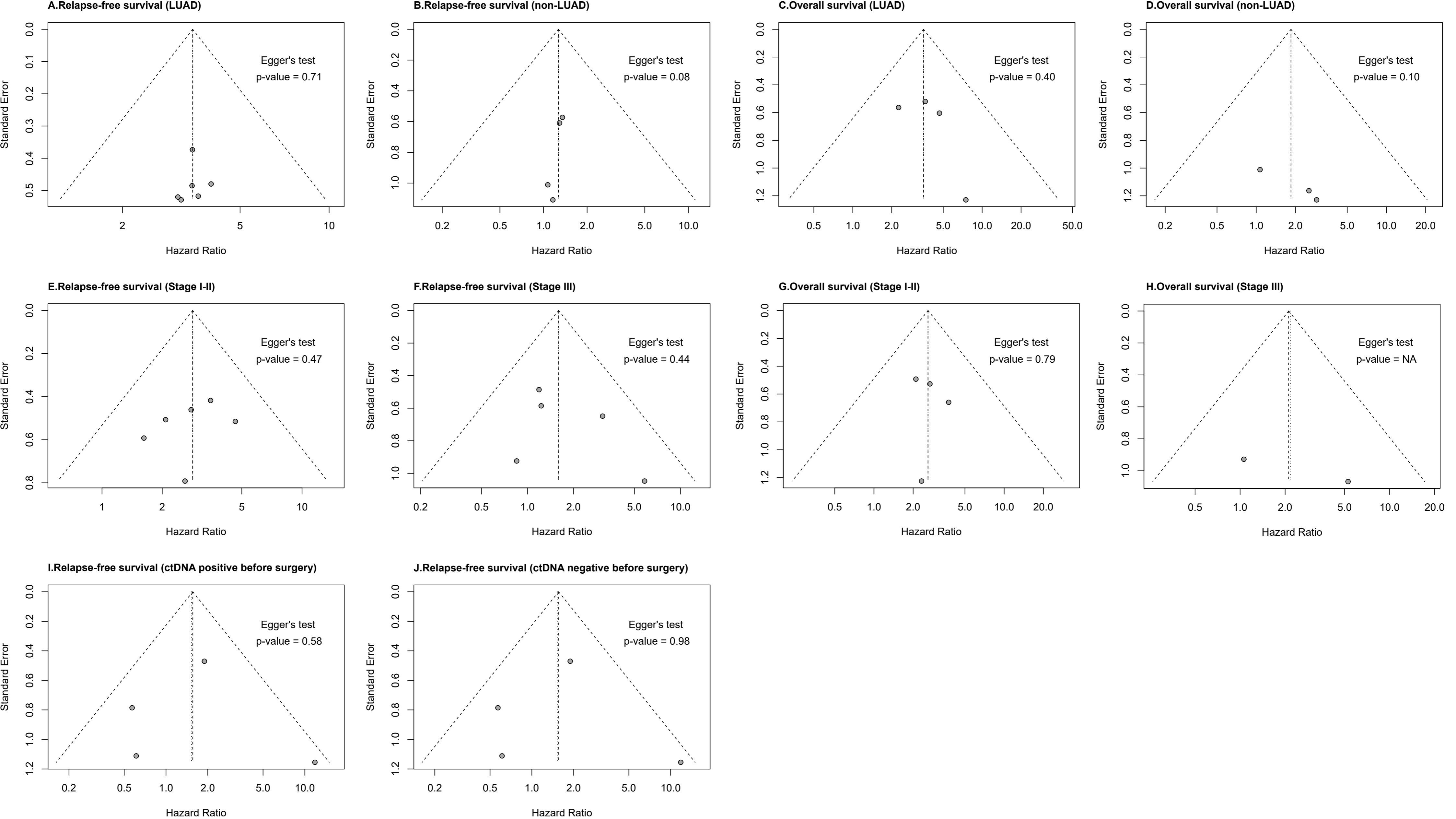

Supplement: Supplementary file 3 — Supplementary file3 (PDF 326 KB) [file 432_2023_5550_MOESM3_ESM.pdf]
