# Supplementary material for: Prognostic value of preoperative circulating tumor DNA in non-small cell lung cancer: a systematic review and meta-analysis
Source: J Cancer Res Clin Oncol. 2024 Jan 22;150(1):25. doi: 10.1007/s00432-023-05550-z (PMC10803397; doi:10.1007/s00432-023-05550-z)

Supplementary Figure 2. Prognostic value of ctDNA (during from the end of NAT to preoperative)

Relapse-free survival (ctDNA after NAT; n = 62)

Study	N	ctDNA detect	ctDNA not detect	Hazard Ratio	HR	95% CI
Yue D 2022	22	20	2		7.41	[0.91; 60.31]
Provencio M 2022	40	13	27		3.85	[1.08; 13.73]
**Overall (Fixed effects)**					**4.59**	**[1.55; 13.61]**
**Overall (Random effects)**					**4.59**	**[1.55; 13.61]**

Heterogeneity: $I^2 = 0\%$, $\tau^2 = 0$, $p = 0.60$

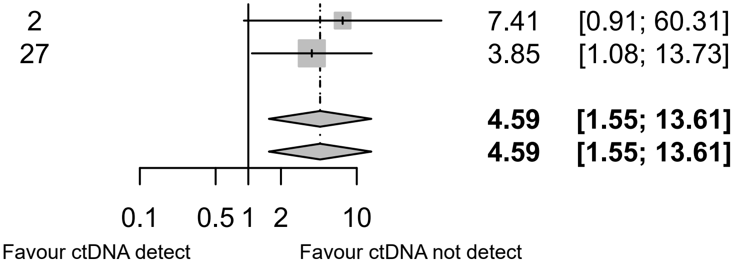

Supplement: Supplementary file 2 — Supplementary file2 (PDF 169 KB) [file 432_2023_5550_MOESM2_ESM.pdf]
