# Supplementary material for: Prognostic value of preoperative circulating tumor DNA in non-small cell lung cancer: a systematic review and meta-analysis
Source: J Cancer Res Clin Oncol. 2024 Jan 22;150(1):25. doi: 10.1007/s00432-023-05550-z (PMC10803397; doi:10.1007/s00432-023-05550-z)
Supplement: Supplementary file 4 — Supplementary file4 (PDF 103 KB) [file 432_2023_5550_MOESM4_ESM.pdf]

Search strategies for all databases

Datebase	Search Keywords	Number
Science Direct	("circulating tumor dna" OR "biopsies liquid" OR "biopsy liquid" OR "liquid biopsies" OR "Neoplasm, Residual") AND ("Carcinoma, Non-Small-Cell Lung" OR Non-Small Cell Lung Cancer) AND ("Prognosis" OR "Survival")	112
PubMed	("neoplasm, residual"[MeSH Terms] OR "MRD"[Title/Abstract] OR "residual neoplasms"[Title/Abstract] OR "residual neoplasm"[Title/Abstract] OR "minimal residual disease"[Title/Abstract] OR "residual minimal disease"[Title/Abstract] OR (("Residual"[All Fields] OR "residuals"[All Fields]) AND "minimal diseases"[Title/Abstract]) OR "residual disease minimal"[Title/Abstract] OR "minimal residual diseases"[Title/Abstract] OR "residual cancer"[Title/Abstract] OR "cancer residual"[Title/Abstract] OR "residual cancers"[Title/Abstract] OR "residual tumor"[Title/Abstract] OR "residual tumors"[Title/Abstract] OR "residual tumour"[Title/Abstract] OR "residual tumours"[Title/Abstract] OR "tumour residual"[Title/Abstract] OR ("circulating tumor dna"[MeSH Terms] OR ("biopsies liquid"[Title/Abstract] OR "biopsy liquid"[Title/Abstract] OR "liquid biopsies"[Title/Abstract] OR "liquid biopsy"[MeSH Terms] OR "circulating tumor dna"[Title/Abstract]))) AND ("Prognosis"[MeSH Terms] OR ("Prognoses"[Title/Abstract] OR "prognostic factors"[Title/Abstract] OR "prognostic"[Title/Abstract] OR "factor prognostic"[Title/Abstract] OR "factors prognostic"[Title/Abstract]) OR "Survival"[Title/Abstract]) AND ("carcinoma, non small cell lung"[MeSH Terms] OR ("carcinoma non small cell lung"[Title/Abstract] OR "carcinomas non small cell lung"[Title/Abstract] OR "lung carcinoma non small cell"[Title/Abstract] OR "lung carcinomas non small cell"[Title/Abstract] OR "non small cell lung carcinomas"[Title/Abstract] OR "non small cell lung carcinoma"[Title/Abstract] OR "non small cell lung carcinoma"[Title/Abstract] OR "carcinoma non small cell lung"[Title/Abstract] OR "non small cell lung carcinoma"[Title/Abstract] OR "non small cell lung cancer"[Title/Abstract] OR "nonsmall cell lung cancer"[Title/Abstract]))	739
Embase	(Carcinoma, Non-Small-Cell Lung.ab. or Carcinoma, Non-Small-Cell Lung.ti. or Carcinoma, Non Small Cell Lung.ab. or Carcinoma, Non Small Cell Lung.ti. or Carcinomas, Non-Small-Cell Lung.ab. or Carcinomas, Non-Small-Cell Lung.ti. or Lung Carcinoma, Non-Small-Cell.ab. or Lung Carcinoma, Non-Small-Cell.ti. or Lung Carcinomas, Non-Small-Cell.ab. or Lung Carcinomas, Non-Small-Cell.ti. or Non-Small-Cell Lung Carcinomas.ab. or Non-Small-Cell Lung Carcinomas.ti. or Non-Small-Cell Lung Carcinoma.ab. or Non-Small-Cell Lung Carcinoma.ti. or Non Small Cell Lung Carcinoma.ab. or Non Small Cell Lung Carcinoma.ti. or Carcinoma, Non-Small Cell Lung.ab. or Carcinoma, Non-Small Cell Lung.ti. or Non-Small Cell Lung Carcinoma.ab. or Non-Small Cell Lung Carcinoma.ti. or Non-Small Cell Lung Cancer.ab. or Non-Small Cell Lung Cancer.ti. or Nonsmall Cell Lung Cancer.ab. or Nonsmall Cell Lung Cancer.ti. or exp non small cell lung cancer/) AND (Circulating Tumor DNA.ab. or Circulating Tumor DNA.ti. or DNA, Circulating Tumor.ab. or DNA, Circulating Tumor.ti. or Tumor DNA, Circulating.ab. or Tumor DNA, Circulating.ti. or Cell-Free Tumor DNA.ab. or Cell-Free Tumor DNA.ti. or Cell Free Tumor DNA.ab. or Cell Free Tumor DNA.ti. or DNA, Cell-Free Tumor.ab. or DNA, Cell-Free Tumor.ti. or Tumor DNA, Cell-Free.ab. or Tumor DNA, Cell-Free.ti. or Liquid Biopsy.ab. or Liquid Biopsy.ti. or Biopsies, Liquid.ab. or Biopsies, Liquid.ti. or Biopsy, Liquid.ab. or Biopsy, Liquid.ti. or Liquid Biopsies.ab. or Liquid Biopsies.ti. or minimal residual disease.ab. or minimal residual disease.ti. or molecular residual disease.ti. or molecular residual disease.ab. or Residual Neoplasms.ab. or Residual Neoplasms.ti. or Minimal Residual Disease.ab. or Minimal Residual Disease.ti. or Residual Cancers.ab. or Residual Cancers.ti. or Tumour, Residual.ab. or Tumour, Residual.ti. or residual minimal disease.ab. or residual minimal disease.ti. or residual disease, minimal.ab. or residual disease, minimal.ti. or residual	1225

	disease.ab. or residual disease.ti. or neoplasm, residual.ab. or neoplasm, residual.ti. or minimum residual disease.ab. or minimum residual disease.ti. or disease, minimal residual.ab. or disease, minimal residual.ti. or exp liquid biopsy/ or exp circulating tumor DNA/ or exp minimal residual disease/) AND (prognosis.ab. or prognosis.ti. or Prognoses.ab. or Prognoses.ti. or Prognostic Factors.ab. or Prognostic Factors.ti. or Prognostic Factor.ab. or Prognostic Factor.ti. or Factor, Prognostic.ab. or Factor, Prognostic.ti. or Factors, Prognostic.ab. or Factors, Prognostic.ti. or Survival.ab. or Survival.ti. or exp survival/ or exp prognosis/)	
Cochrane	(MeSH descriptor: [Carcinoma, Non-Small-Cell Lung] explode all trees OR (Carcinoma, Non Small Cell Lung):ti,ab,kw OR (Carcinomas, Non-Small-Cell Lung):ti,ab,kw OR (Lung Carcinoma, Non-Small-Cell):ti,ab,kw OR (Lung Carcinomas, Non-Small-Cell):ti,ab,kw OR (Non-Small-Cell Lung Carcinomas):ti,ab,kw OR (Nonsmall Cell Lung Cancer):ti,ab,kw, OR (Non-Small-Cell Lung Carcinoma):ti,ab,kw OR (Non Small Cell Lung Carcinoma):ti,ab,kw OR (Carcinoma, Non-Small Cell Lung):ti,ab,kw OR (Non-Small Cell Lung Carcinoma):ti,ab,kw OR (Non-Small Cell Lung Cancer):ti,ab,kw) AND (MeSH descriptor: [Prognosis] explode all trees OR (prognoses):ti,ab,kw OR (Prognostic Factors):ti,ab,kw OR (Prognostic Factor):ti,ab,kw OR (Factor, Prognostic):ti,ab,kw OR (Factors, Prognostic):ti,ab,kw OR (Survival):ti,ab,kw)) AND (MeSH descriptor: [Circulating Tumor DNA] explode all trees OR (biopsies liquid):ti,ab,kw OR (biopsy liquid):ti,ab,kw OR (liquid biopsies):ti,ab,kw OR MeSH descriptor: [Liquid Biopsy] explode all trees OR (ctDNA):ti,ab,kw OR (MeSH descriptor: [Neoplasm, Residual] explode all trees) OR (Residual Neoplasms):ti,ab,kw OR (Residual Neoplasm):ti,ab,kw OR (Minimal Residual Disease):ti,ab,kw OR (Minimal Disease, Residual):ti,ab,kw OR (Residual Minimal Disease):ti,ab,kw OR (Residual Disease, Minimal):ti,ab,kw, OR (Residual Cancer):ti,ab,kw OR (Cancer, Residual):ti,ab,kw OR (Residual Cancers):ti,ab,kw OR (Residual Tumors):ti,ab,kw OR (Residual Tumour):ti,ab,kw, OR (Residual Tumours):ti,ab,kw OR (Tumour, Residual):ti,ab,kw)	237
