# Supplementary material for: Prognostic value of preoperative circulating tumor DNA in non-small cell lung cancer: a systematic review and meta-analysis
Source: J Cancer Res Clin Oncol. 2024 Jan 22;150(1):25. doi: 10.1007/s00432-023-05550-z (PMC10803397; doi:10.1007/s00432-023-05550-z)
Supplement: Supplementary file 5 — Supplementary file5 (PDF 713 KB) [file 432_2023_5550_MOESM5_ESM.pdf]

Prognostic Value of Preoperative Circulating Tumor DNA in Non-Small Cell Lung Cancer:

A Systematic Review and Meta-analysis

Summary of supplementary tables

**Table S1** Definition of endpoints for studies included in the meta-analysis.
**Table S2** Results of quality assessment using the Newcastle-Ottawa Scale for cohort studies.
**Table S3** Univariate regression analysis.
**Table S4** Multivariate regression analysis.

Table S1. Definition of endpoints for studies included in the meta-analysis

Author	Year	Endpoint definitions
Peng M	2020	RFS：defined as the length of time after surgery that the patient survives without any signs or symptoms of lung cancer.<br>OS：defined as the length of time from the date of diagnosis until death.
Qiu B	2021	RFS：measured from the date of surgery to the verified first radiographic recurrence (local or distant).
Tan A	2021	NA
Waldeck S	2022	PFS：defined as time from blood sample collection to any of the following: disease progression as defined by RECIST 1.1-based radiographic assessment, death from any cause or last follow-up visit.<br>OS：considered as secondary endpoint and was defined as the time from blood sample collection to death from any cause or last follow up.
Xia L	2022	RFS：defined as the time interval from the surgery to the first verified recurrence (local or distant) or death for any cause.
Gale, D	2022	RFS：measured from the date of surgery to the events either recurrence of the first primary tumour, or death if not preceded by a second primary tumour<br>OS：measured from the date of surgery to the death for any cause
Li, N	2022	RFS：measured from the date of surgery to the first documented radiologic recurrence/metastasis as a result of NSCLC<br>OS：measured from the date of surgery to the death as a result of NSCLC
Yue, D	2022	RFS：defined as the time between the date of surgery and the date when local recurrence or distant relapses were diagnosed
Provencio, M	2022	OS：defined as the time from the start of neoadjuvant treatment to death from any cause.<br>PFS：defined as the time between the start of neoadjuvant treatment and disease progression
Zhang, JT	2022	DFS：measured from the day of definitive surgery to the first radiographic recurrence or death.
Chen, KZ	2022	OS: estimated from the date of surgical resection until death of any cause or the date of the last follow-up<br>DFS: defined as the time from the day of surgery until the first event (relapse or metastasis) or last follow-up

Table S2. Results of quality assessment using the Newcastle-Ottawa Scale for cohort studies

Study	Selection				Comparability	Outcome			Scores
	Representativeness of the exposed cohort	Selection of the non exposed cohort	Ascertainment of exposure	Demonstration that outcome of interest was not present at start of study	Comparability of cohorts on the basis of the design or analysis*	Assessment of outcome	Was follow-up long enough for outcomes to occur	Adequacy of follow up of cohorts	
Peng M 2020	☆	☆	☆	☆	☆	☆	☆	☆	8
Qiu B 2021	☆	☆	☆	☆	☆	☆		☆	7
Tan A 2021	☆	☆	☆		☆		☆		5
Waldeck S 2022		☆	☆	☆	☆	☆	☆	☆	7
Xia L 2022	☆	☆	☆	☆	☆☆	☆	☆	☆	9
Gale, D 2022	☆	☆	☆	☆	☆	☆	☆	☆	8
Li, N 2022	☆	☆	☆	☆	☆	☆	☆	☆	8
Yue, D 2022		☆	☆	☆	☆	☆		☆	6
Provencio, M 2022	☆	☆	☆	☆	☆☆	☆	☆	☆	9
Zhang, JT 2022		☆	☆	☆	☆	☆	☆	☆	7
Chen, KZ 2022	☆	☆	☆	☆	☆	☆	☆	☆	8

* A maximum of 2 stars can be allotted in this category, one for pre-surgery ctDNA, the other for other controlled factors.

Table S3.Univariate regression analysis

RFS: Pre-surgery ctDNA presence in patients.

Study	HR	95%CI
TAN A 2021	5.46	1.21-24.62
Zhang JT 2022	4.85	2.63-8.94
Waldeck S 2022	2.00	0.41-9.78
Yue D 2022	7.41	0.91-60.28
Xia L 2022	4.19	2.61-6.72
Peng M 2020	3.72	1.61-8.60
Qiu B 2021	2.58	1.00-6.67
Li N 2022	2.44	1.12-5.32
Gale D 2022	3.08	1.39-6.82
Chen KZ 2022	2.79	1.13-6.88
Overall (Fixed effects)	3.62	2.80-4.67
Overall (Random effects)	3.62	2.80-4.67
Heterogeneity: I2 = 0%, τ2 = 0, p = 0.88		

OS: Pre-surgery ctDNA presence in patients.

Study	HR	95%CI
Waldeck S 2022	3.01	0.31-29.32
Peng M 2020	4.91	1.68-14.37
Li N 2022	5.54	1.01-30.38
Gale D 2022	2.76	1.14-6.68
Chen KZ 2022	2.10	0.80-5.52
Overall (Fixed effects)	3.12	1.86-5.23
Overall (Random effects)	3.12	1.86-5.23
Heterogeneity: I2 = 0%, τ2 = 0, p = 0.76		

RFS: Pre-surgery ctDNA presence in LUAD patients.

Study	HR	95%CI
Xia L 2022	6.02	3.42-10.59
Peng M 2020	3.44	1.33-8.90
Qiu B 2021	3.61	1.31-9.95
Li N 2022	3.16	1.12-8.91
Gale D 2022	3.99	1.56-10.22
Chen KZ 2022	3.08	1.11-8.54
Overall (Fixed effects)	4.27	3.02-6.05
Overall (Random effects)	4.27	3.02-6.05
Heterogeneity: I2 = 0%, τ2 = 0, p = 0.78		

OS: Pre-surgery ctDNA presence in LUAD patients.

Study	HR	95%CI
Peng M 2020	4.68	1.43-15.29
Li N 2022	7.48	0.67-83.24
Gale D 2022	3.63	1.31-10.06
Chen KZ 2022	2.26	0.75-6.82
Overall (Fixed effects)	3.52	1.91-6.49
Overall (Random effects)	3.52	1.91-6.49
Heterogeneity: I^2 = 0%, τ^2 = 0, p = 0.75		

RFS: Pre-surgery ctDNA presence in non-LUAD patients.

Study	HR	95%CI
Waldeck S 2022	1.16	0.13-10.20
Xia L 2022	1.35	0.44-4.15
Li N 2022	1.29	0.39-4.26
Chen KZ 2022	1.07	0.15-7.76
Overall (Fixed effects)	1.27	0.62-2.59
Overall (Random effects)	1.27	0.62-2.59
Heterogeneity: $I^2 = 0\%$, $\tau^2 = 0$, $p = 1.00$		

OS: Pre-surgery ctDNA presence in non-LUAD patients.

Study	HR	95%CI
Waldeck S 2022	2.54	0.26-24.81
Li N 2022	2.91	0.26-32.37
Chen KZ 2022	1.07	0.15-7.76
Overall (Fixed effects)	1.85	0.52-6.59
Overall (Random effects)	1.85	0.52-6.59
Heterogeneity: $I^2 = 0\%$, $\tau^2 = 0$, $p = 0.78$		

RFS: Pre-surgery ctDNA presence in stage I-II patients.

Study	HR	95%CI
Xia L 2022	5.00	2.85-8.78
Peng M 2020	2.69	1.03-7.02
Qiu B 2021	2.60	0.55-12.28
Li N 2022	1.62	0.51-5.17
Gale D 2022	3.17	1.29-7.78
Chen KZ 2022	2.79	1.13-6.88
Overall (Fixed effects)	3.39	2.37-4.84
Overall (Random effects)	3.30	2.24-4.85
Heterogeneity: $I^2 = 0\%$, $\tau^2 = 0.0238$, $p = 0.56$		

OS: Pre-surgery ctDNA presence in stage I-II patients.

Study	HR	95%CI
Peng M 2020	3.75	1.03-13.65
Li N 2022	2.32	0.21-25.59
Gale D 2022	2.69	0.96-7.58
Chen KZ 2022	2.1	0.80-5.52
Overall (Fixed effects)	2.60	1.43-4.74
Overall (Random effects)	2.60	1.43-4.74
Heterogeneity: $I^2 = 0\%$, $\tau^2 = 0$, $p = 0.92$		

RFS: Pre-surgery ctDNA presence in stage III patients.

Study	HR	95%CI
Xia L 2022	1.60	0.66-3.88
Peng M 2020	5.83	0.75-45.43
Qiu B 2021	2.13	0.63-7.20
Li N 2022	1.23	0.39-3.87
Gale D 2022	0.85	0.14-5.20
Overall (Fixed effects)	1.65	0.95-2.88
Overall (Random effects)	1.65	0.95-2.88
Heterogeneity: $I^2 = 0\%$, $\tau^2 = 0$, $p = 0.66$		

OS: Pre-surgery ctDNA presence in stage III patients.

Study	HR	95%CI
Peng M 2020	5.27	0.65-42.72
Gale D 2022	1.06	0.17-6.53
Overall (Fixed effects)	2.11	0.54-8.34
Overall (Random effects)	2.17	0.45-10.34
Heterogeneity: $I^2 = 22\%$, $\tau^2 = 0.2855$, $p = 0.26$		

RFS: Pre-surgery ctDNA presence in patients (postoperative AT).

Study	HR	95%CI
Xia L 2022	0.35	0.17-0.73
Qiu B 2021	0.49	0.22-1.10
Gale D 2022	0.67	0.19-2.36
Overall (Fixed effects)	0.44	0.27-0.72
Overall (Random effects)	0.44	0.27-0.72
Heterogeneity: $I^2 = 0\%$, $\tau^2 = 0$, $p = 0.64$		

RFS: Pre-surgery ctDNA not presence in patients (postoperative AT).

Study	HR	95%CI
Xia L 2022	3.58	1.88-6.82
Qiu B 2021	0.61	0.07-5.46
Gale D 2022	11.82	1.23-113.64
Waldeck S 2022	0.57	0.12-2.61
Overall (Fixed effects)	2.70	1.55-4.70
Overall (Random effects)	1.98	0.56-7.05
Heterogeneity: $I^2 = 63\%$, $\tau^2 = 1.0053$, $p = 0.04$		

***RFS: relapse-free survival. OS: overall survival. AT: adjuvant therapy**

Table S4. Multivariate regression analysis

RFS: Pre-surgery ctDNA presence in patients.

Study	HR	95%CI
Xia L 2022	2.6	1.31-5.15
Peng M 2020	3.46	1.37-8.71
Qiu B 2021	3.03	1.04-8.82
Li N 2022	1.33	0.58-3.07
Gale D 2022	2.22	0.9-5.48
Provencio M 2022	3.85	1.08-13.73
Overall (Fixed effects)	2.45	1.70-3.53
Overall (Random effects)	2.45	1.70-3.53
Heterogeneity: $I^2 = 0\%$, $\tau^2 = 0$, $p = 0.65$		

OS: Pre-surgery ctDNA presence in patients.

Study	HR	95%CI
Peng M 2020	3.39	1.28-8.97
Gale D 2022	1.75	0.68-4.53
Provencio M 2022	23	1.84-287.72
Overall (Fixed effects)	2.81	1.46-5.42
Overall (Random effects)	3.03	1.31-7.00
Heterogeneity: $I^2 = 47\%$, $\tau^2 = 0.1644$, $p = 0.15$		

RFS: Pre-surgery ctDNA presence in LUAD patients.

Study	HR	95%CI
Xia L 2022	3.45	1.66-7.18

OS: Pre-surgery ctDNA presence in LUAD patients.

Study	HR	95%CI
NA	NA	NA

RFS: Pre-surgery ctDNA presence in non-LUAD patients.

Study	HR	95%CI
NA	NA	NA

OS: Pre-surgery ctDNA presence in non-LUAD patients.

Study	HR	95%CI
NA	NA	NA

RFS: Pre-surgery ctDNA presence in stage I-II patients.

Study	HR	95%CI
Xia L 2022	3.49	1.54-7.92
Peng M 2020	2.08	0.77-5.62
Gale D 2022	4.64	1.69-12.72
Overall (Fixed effects)	3.25	1.90-5.56
Overall (Random effects)	3.25	1.90-5.56
Heterogeneity: $I^2 = 0\%$, $\tau^2 = 0$, $p = 0.53$		

OS: Pre-surgery ctDNA presence in stage I-II patients.

Study	HR	95%CI
NA	NA	NA

RFS: Pre-surgery ctDNA presence in stage III patients.

Study	HR	95%CI
Xia L 2022	1.19	0.46-3.09
Qiu B 2021	3.1	0.87-11.06
Overall (Fixed effects)	1.68	0.78-3.60
Overall (Random effects)	1.74	0.70-4.37
Heterogeneity: $I^2 = 28\%$, $\tau^2 = 0.1300$, $p = 0.24$		

OS: Pre-surgery ctDNA presence in stage III patients.

Study	HR	95%CI
NA	NA	NA

RFS: Pre-surgery ctDNA presence in patients (postoperative AT).

Study	HR	95%CI
Xia L 2022	0.24	0.1-0.58
Qiu B 2021	0.47	0.2-1.11
Overall (Fixed effects)	0.34	0.18-0.63
Overall (Random effects)	0.34	0.18-0.65
Heterogeneity: $I^2 = 13\%$, $\tau^2 = 0.0297$, $p = 0.28$		

RFS: Pre-surgery ctDNA not presence in patients (postoperative AT).

Study	HR	95%CI
Xia L 2022	1.89	0.75-4.74

***RFS: relapse-free survival. OS: overall survival. AT: adjuvant therapy**
